# Cost-Utility Analysis of Add-on Cannabidiol vs Usual Care Alone for the Treatment of Seizures in Patients With Treatment-Resistant Lennox-Gastaut Syndrome or Dravet Syndrome in the Netherlands

**DOI:** 10.36469/001c.126071

**Published:** 2024-12-23

**Authors:** Jamshaed Siddiqui, Sally Bowditch

**Affiliations:** 1 FIECON, London, UK; 2 Jazz Pharmaceuticals UK Ltd., London, UK

**Keywords:** cannabidiol, Lennox-Gastaut syndrome, Dravet syndrome, Netherlands, cost-effectiveness, seizures

## Abstract

**Background:** Lennox-Gastaut syndrome (LGS) and Dravet syndrome (DS) are severe, treatment-refractory, epileptic encephalopathies that often develop in infancy or early childhood. Since December 1, 2022, plant-derived highly purified cannabidiol (CBD) medicine (Epidyolex®; 100 mg/mL oral solution) has been reimbursed in the Netherlands for the adjunctive treatment of seizures associated with LGS or DS. **Objective:** To estimate the cost-effectiveness of CBD plus usual care vs usual care alone in patients with LGS or DS in the Netherlands. **Methods:** A cohort-based Markov model from a Dutch societal perspective, based on seizure frequency and seizure-free days, was developed for patients receiving CBD plus usual care (antiseizure medications, including clobazam) or usual care alone. Population characteristics, clinical inputs, and utility values were sourced from CBD clinical trials and quality-of-life studies. Drug acquisition, disease management, adverse events, and societal costs from published literature were included. A 2019/2020 price year in euros was used. The model used a mean dosage of 12 mg/kg/day, a lifetime (90-year) horizon, and a 3-month cycle length. Discount rates of 4.0% and 1.5% per annum were applied to costs and outcomes, respectively. Uncertainty was explored through deterministic and probabilistic sensitivity analyses. **Results:** In patients with LGS, CBD plus usual care led to additional costs of €28 338 and increased quality-adjusted life-years (QALYs) of 1.318 compared with usual care alone. The incremental cost-effectiveness ratio of €21 493/QALY in LGS is below the willingness-to-pay threshold of €80 000/QALY in the Netherlands. In patients with DS, CBD plus usual care dominated usual care alone, with cost savings of €23 642 and increased QALYs of 0.868. The probability that CBD plus usual care is cost-effective in the Netherlands compared with usual care alone is 96% and 99% in patients with LGS and DS, respectively. **Discussion:** Elicitation methods were used to address data gaps in model inputs (eg, healthcare resource utilization and utilities); Dutch clinical experts, sensitivity, and scenario analyses validated this approach. **Conclusions:** Based on a willingness-to-pay threshold of €80 000, the base case cost-utility analysis demonstrated the cost-effectiveness of CBD plus usual care in patients with treatment-refractory LGS or DS aged 2 years or older in the Netherlands.

## INTRODUCTION

Lennox-Gastaut syndrome (LGS) and Dravet syndrome (DS) are severe, treatment-refractory, lifelong epileptic encephalopathies that often develop in infancy or early childhood.^1,2^ LGS is associated with multiple types of generalized, treatment-resistant seizures paired with an interictal electroencephalogram showing diffuse slow spike-and-wave activity, generalized paroxysmal bursts, and a slow background.^1,3^ Seizure onset in LGS often occurs in childhood (age <8 years),^1,3^ and there is an estimated prevalence of 3 to 28 per 100 000 people for a confirmed/narrow definition of LGS.^4^ Conversely, DS is distinguished by prolonged, febrile, afebrile, focal clonic, or generalized clonic seizures^5^ that are often triggered by fever or illness in the first year of life.^2^ In Europe, DS is less common than LGS, with a prevalence of approximately 7 per 100 000 people.^5^

Patients with LGS or DS have an increased risk of mortality, and both syndromes have a significant impact on the quality of life (QoL) of patients, families, and caregivers.^6–8^ Patients with LGS experience a high seizure burden, with at least half of patients suffering from sudden drop seizures,^1,7^ which can lead to progressive dysfunction of the brain with associated cognitive impairment, developmental delays, and behavioral disturbances that can prevent patients from achieving independence in adult life.^1,9^ Similarly to LGS, patients with DS exhibit the hallmarks of developmental epileptic encephalopathies with a high seizure burden, particularly before the age of 5 years, which impacts normal development and cognition.^5^ Patients with LGS and DS are at high risk of sudden unexpected death in epilepsy (SUDEP).^10,11^ Compared with children without epilepsy, the all-cause risk of mortality is 3 times higher in children with epilepsy and 14 times higher for children with LGS.^12^ In children with DS, epilepsy-related mortality by 18 years of age is estimated at 7%,^13^ with 32% of deaths caused by status epilepticus and 49% by SUDEP at all ages.^11^

Based on a 2020 report on the population in the Netherlands,^14^ and literature on the prevalence of LGS and DS in other countries,^15,16^ the number of patients with LGS and DS in the Netherlands was calculated as approximately 2000 and 600, respectively. Despite the various treatment options available for these patients, including several approved antiseizure medications (ASMs), seizure control is unachievable in the majority of patients.^17^ The treatment-refractory nature of these syndromes leads to a substantial healthcare-associated economic burden arising from ASM costs, general practitioner and outpatient visits, laboratory services, physiotherapy, hospitalization, emergency department visits, and home-based or institutionalized care.^4,18^ There remains a significant unmet need in these life-threatening syndromes for treatments that reduce seizure frequency and increase the number of seizure-free days. A reduction in these outcomes has been shown to be associated with an improvement in QoL for both patients and caregivers.^19,20^

Highly purified cannabidiol (CBD; Epidyolex®) was approved by the European Medicines Agency (EMA) on September 19, 2019, for the adjunctive treatment of seizures associated with LGS or DS, in conjunction with clobazam, for patients 2 years of age and older.^21^ CBD has been robustly evaluated for use in patients with LGS or DS in the following global randomized controlled trials (RCTs): GWPCARE3 (NCT02224560) and GWPCARE4 (NCT02224690) for LGS; GWPCARE1 (NCT02091206, NCT02091375) and GWPCARE2 (NCT02224703) for DS.^22–25^ Furthermore, these trials are supported by an open-label extension (OLE) for CBD, GWPCARE5 (NCT02224573).^26,27^ In the RCTs and OLE, CBD demonstrated sustained efficacy over 3 years, with an acceptable safety profile, in patients with LGS or DS.^22–27^ In addition to reducing the frequency of seizures, CBD treatment was also associated with seizure freedom in some patients.^22–27^ This study was conducted with Epidiolex® (US)/Epidyolex® (EU), and results do not apply to other CBD-containing products.

Since December 1, 2022, CBD has been reimbursed in the Netherlands for patients with LGS or DS in line with the EMA approval.^21,28^ The objective of this study was to perform a cost-utility analysis of CBD plus usual care compared with usual care alone in patients with LGS or DS in the Netherlands, to help inform treatment decisions by patients, caregivers, and clinicians.

## METHODS

A cohort-based Markov model was developed in Microsoft Excel to analyze the costs and consequences of treatment with CBD plus usual care compared with usual care alone for seizures in patients with treatment-resistant LGS or DS. In this model, usual care is defined as the established clinical management for each condition, including clobazam, alongside other ASMs. Analysis was conducted from a societal perspective in the Netherlands with a lifetime horizon of 90 years to reflect the lifelong nature of these syndromes, and to align with previous CBD cost-effectiveness studies in LGS and DS.^29–32^ Costs and quality-adjusted life years (QALYs) were discounted annually at rates of 4.0% and 1.5%, respectively, as recommended by the Zorginstituut Nederland (ZiN) economic evaluations in healthcare guidelines.^33^ The model used in the study is not publicly available owing to the confidentiality of the data. Given that this study followed the ZiN guidelines, a health economic analysis plan was not developed.

### Patient Population

In line with the marketing authorization for CBD,^21^ this cost-effectiveness model included patients with LGS or DS aged 2 years or older in whom the conditions were inadequately controlled by established usual care. Data included in the model were derived from patients treated with highly purified CBD (Epidyolex®; 100 mg/mL oral solution) who completed the RCTs (GWPCARE1-4)^22–25^ and entered the OLE (GWPCARE5).^26,27^ Eligible patients with LGS were those with a history of slow (<3 Hz) spike-and-wave pattern electroencephalogram recordings and 2 or more drop seizures per week during the baseline period of the parent trial.^26^ Patients with DS were eligible if they had 4 or more convulsive seizures in the baseline period of the parent trial.^27^ The RCTs and OLE were conducted in patients with LGS or DS recruited from Australia, Europe, and/or the United States, with GWPCARE2, GWPCARE4, and GWPCARE5, including patients from the Netherlands.^22–27^ The key characteristics of patients included in this model were validated by clinical experts, who confirmed that they are generalizable to patients within Dutch clinical practice.

### Comparator

This model focused on CBD as a last-line add-on treatment option in patients who have not achieved sufficient seizure control with established usual care. Given the rarity of LGS and DS, the heterogeneity of patients, and the variety of available ASMs, it is not clinically or statistically meaningful to compare the intervention with individual or specific combinations of ASMs. Therefore, the model compared add-on CBD plus usual care, consisting of ASMs including clobazam, with usual care alone. ASMs included within usual care for patients with LGS or DS are summarized in **Table 1**, based on data from the RCTs and the subgroup of patients taking clobazam as per the license.^21^

**Table 1.** Model Inputs for Patients With Lennox-Gastaut Syndrome and Dravet Syndrome

	**Lennox-Gastaut Syndrome**				**Dravet Syndrome**			
**Demographic characteristics at baseline (n = 10 000)^a^**								
**Age group, y**	**2–5**	**6–11**	**12–17**	**18–55**	**2–5**	**6–11**	**12–17**	**18–55**
Proportion of patients, % (SE)	13 (0.03)	36 (0.07)	25 (0.05)	26 (0.05)	30 (0.06)	39 (0.08)	29 (0.06)	2 (0.00)
Mean weight, kg (SE)	17.9 (0.87)	28.7 (1.34)	51.4 (2.67)	59.7 (2.65)	18.2 (0.53)	30.5 (1.14)	55.0 (2.27)	49.3 (2.27)
**Age group**	**≥2 years**				**≥2 years**			
Mean age, y (SE)	13.2 (2.63)				11.5 (2.30)			
**Health state allocation at baseline: No. of seizures per 3 months**	**≤55 drop seizures**		**>55 drop seizures**		**≤12 convulsive seizures**		**>12 convulsive seizures**	
Proportion of patients, % (SE)	38 (0.08)		62 (0.12)		50 (0.10)		50 (0.10)	
**Sub-health state allocation at baseline: No. of days without seizures per 3 months**	**No. of days without drop seizures per month (SE)**				**No. of days without convulsive seizures per month (SE)**			
	**≤15 days**	**>15 days**	**≤15 days**	**>15 days**	**≤18 days**	**>18 days**	**≤18 days**	**>18 days**
Proportion of patients, % (SE)	81 (0.16)	19 (0.04)	99 (0.20)	1 (0.00)	1 (0.00)	99 (0.20)	79 (0.16)	21 (0.04)
**Resource use, %**								
**ASM**	**Value**	**SE**	**Lower bound**	**Upper bound**	**Value**	**SE**	**Lower bound**	**Upper bound**
Clobazam	100	0.20	100.00	100.00	100	0.20	100.00	100.00
Clonazepam	5	0.01	3.27	7.22	7	0.01	4.20	9.30
Levetiracetam	35	0.07	21.88	49.04	20	0.04	12.83	28.54
Rufinamide	28	0.06	17.79	39.71	5	0.01	3.16	6.98
Stiripentol	0	0.00	0.00	0.00	44	0.09	27.26	61.51
Topiramate	14	0.03	9.00	19.97	22	0.04	13.85	30.83
Valproate sodium	0	0.00	0.00	0.00	22	0.04	13.85	30.83
Valproic acid	24	0.05	15.36	34.22	64	0.13	37.46	86.69
Zonisamide	12	0.03	7.94	17.59	11	0.02	6.99	15.48
**Cost of care and hours of care per week^b^**								
	**Health state**		**Age group of patients with LGS**		**Health state**		**Age group of patients with DS**	
			**2-17 y**	**18-55 y**			**2-17 y**	**18-55 y**
Hours of family care per week (SE) (€14.95 [replacement cost] per hour)	Seizure-free		34.0 (6.8)	6.8 (1.4)	Seizure-free		30.9 (6.2)	6.2 (1.2)
	≤55 seizures		67.5 (13.5)	13.5 (2.7)	≤12 seizures		61.3 (12.3)	12.3 (2.5)
	>55 seizures		72.1 (14.4)	14.4 (2.9)	>12 seizures		66.1 (13.2)	13.2 (2.6)
Hours of professional care per week (SE) (€53.40 per hour)	Seizure-free		22.7 (4.5)	4.5 (0.9)	Seizure-free		20.6 (4.1)	4.1 (0.8)
	≤55 seizures		45.0 (9.0)	9.0 (1.8)	≤12 seizures		40.9 (8.2)	8.2 (1.6)
	>55 seizures		48.1 (9.6)	9.6 (1.9)	>12 seizures		44.1 (8.8)	8.8 (1.8)
**Utility values^c^**								
**No. of seizures per month**	**No. of days without drop seizures per month (SE)**				**No. of days without convulsive seizures per month (SE)**			
	**≤15 days**		**>15 days**		**≤18 days**		**>18 days**	
Seizure-free	N/A		0.754 (0.037)		N/A		0.778 (0.023)	
≤55 drop seizures (LGS); ≤12 convulsive seizures (DS)	0.375 (0.000)		0.565 (0.047)		0.000 (0.000)		0.690 (0.028)	
>55 drop seizures (LGS); >12 convulsive seizures (DS)	−0.037 (0.062)		0.153 (0.063)		0.267 (0.053)		0.437 (0.000)	

Abbreviations: ASM, antiseizure medication; DS, Dravet syndrome; LGS, Lennox-Gastaut syndrome; N/A, not applicable; SE, standard error.

^a^Model inputs are based on data from the randomized clinical trials in Lennox-Gastaut syndrome and Dravet syndrome. The number of patients relate to the modeled cohort.

^b^Hours of care are based on a caregiver burden survey conducted in Sweden and validated by Dutch clinical experts.

^c^Utility data are based on information from a time trade-off vignette study conducted between March and April 2020.^34^ This study involved interviews with participants from the general populations of the UK and Sweden, using a combination of online and face-to-face methods; 100 participants evaluated each of the 6 patient and caregiver vignettes for LGS and DS (total of 200 participants and 12 vignettes), and they were recruited and interviewed by 5 interviewers trained in the time trade-off ping-pong method.

### CBD Dosage

The base-case analysis used an average CBD dosage of 12 mg/kg/day based on the anticipated average CBD dosage prescribed to patients in a real-world setting in Europe. According to the EMA Summary of Product Characteristics, the recommended starting dosage of CBD is 5 mg/kg/day, which should be increased to the maintenance dosage of 10 mg/kg/day after 1 week.^21^ However, the dosage can be further increased to a maximum recommended dosage of 20 mg/kg/day based on individual clinical response and tolerability.^21^ The model used an average CBD dosage of 12 mg/kg/day to reflect the average dosage of CBD taken by patients in clinical practice in Europe, given that around 20% of patients are expected to increase the maintenance dosage of CBD of 10 mg/kg/day to a maximum dosage of 20 mg/kg/day.^21^ During interviews, Dutch clinical experts indicated that in their clinical practice, most patients with LGS or DS are treated with a CBD dosage below the maximum recommended dosage of 20 mg/kg/day, with an average dosage of approximately 12 mg/kg/day. This average dosage also aligns with real-world data on the use of CBD in Germany,^35^ and was subsequently requested and accepted by the ZiN for decision-making purposes. Furthermore, an average CBD dosage of 12 mg/kg/day was considered acceptable by the National Institute for Health and Care Excellence (NICE) in the appraisals of add-on CBD in LGS and DS.^29,30^

### Model Overview

**Figure 1.**
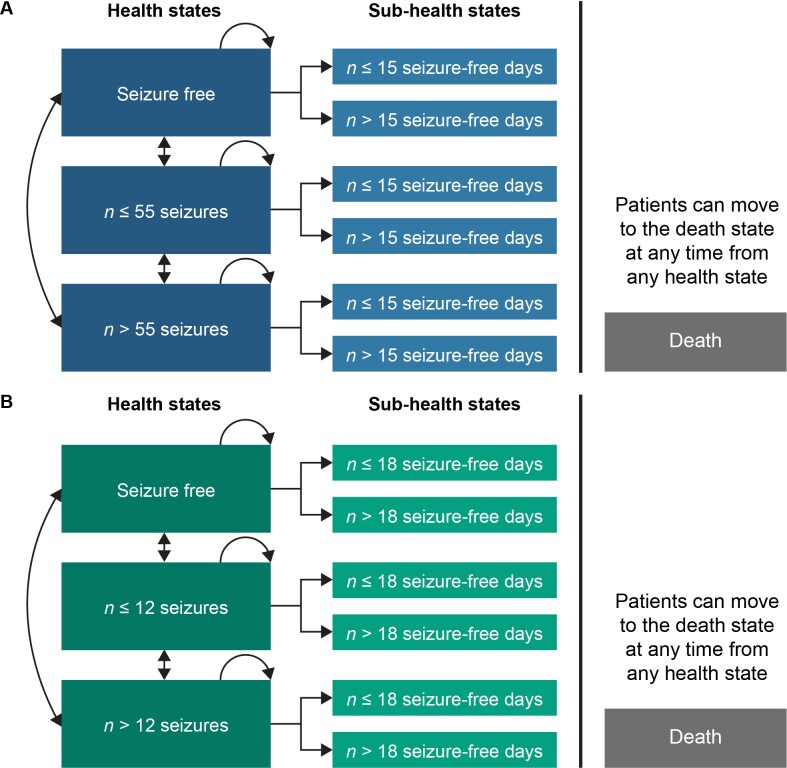
Markov Model Structure for Lennox-Gastaut Syndrome (**A**) and Dravet Syndrome (**B**) Abbreviations: DS, Dravet syndrome; LGS, Lennox-Gastaut syndrome; n, number.

A cohort-based Markov model that captures the major characteristics and natural history of LGS and DS in the Netherlands was developed. This was preferred to a microsimulation model, as analysis of patient-level data (PLD) from the RCTs for LGS and DS showed no significant difference in treatment effect across patient subgroups (eg, stratified by age, sex, or the number or type of previous ASMs). The model determined the average costs and QALYs per patient associated with changes in seizure frequency and seizure-free days, for patients treated with CBD plus usual care compared with usual care alone (**Figure 1**). Underlying assumptions and justifications of the model are detailed in **Table S1** (see **Online Supplementary Material**). The suitability of using seizure frequency and seizure-free days as health states in cohort-based Markov models was endorsed by NICE and the Scottish Medicines Consortium (SMC) appraisals of add-on CBD in LGS and DS. The NICE appraisal was conducted in 2019 on behalf of the National Health Service (NHS) in England and Wales^29,30^ and the SMC appraisal in 2020 for NHS Scotland.^31,32^ Furthermore, the model structure and health state definitions were validated by Dutch clinical experts. The effect of treatment on society was also modeled indirectly based on changes in seizure frequency. The upper limit willingness-to-pay (WTP) threshold in the Netherlands of €80 000/QALY was used in this analysis. Based on the disease burden of LGS and DS, the upper WTP threshold is appropriate for cost-effectiveness analyses in these conditions.^36,37^

The models for LGS and DS were identical except for the health state and sub-health state thresholds, which were selected to provide a balance of patients in each health state.

Overall, 20 000 patients with LGS or DS entered the model via any one of the two health states based on seizure frequency (**Figure 1**). As the RCTs for LGS and DS focused on recruiting patients with treatment-resistant seizures, no patients had seizure-free periods at baseline. Improvements in patients’ QoL have been linked to both the reduction in the total number of seizures and an increase in the number of seizure-free days.^20,38^ Therefore, each seizure frequency health state was categorized into 2 subcategories based on the number of seizure-free days experienced (**Figure 1**). Given that patients with a low seizure frequency are more likely to experience a high number of seizure-free days compared with patients with a high seizure frequency, these subcategories help in assigning different utility scores for patients in a specific seizure group based on the number of seizure-free days experienced. The model used a cycle length of 3 months to align with the treatment follow-up periods in the RCTs.^22–25^

The clinical data used to inform the baseline characteristics, transition probabilities, safety, short-term discontinuation rates, and stopping rule rates were informed by the subpopulation of all randomized patients receiving clobazam as per the license^21^ in the CBD 10 mg/kg/day and 20 mg/kg/day arms (CBD plus usual care) or the placebo arm of the RCTs (usual care alone), who carried over into the OLE. Data on the transition probabilities are not included owing to the confidentiality of the data. Mid-term discontinuation rates were informed by clinical data from the OLE. Data from the intent-to-treat population were not used since the model focused only on the patient population specified in the European license.^21^

### Baseline Characteristics

Baseline characteristics for patients entering the model included age, weight, number of seizures, and seizure-free days (**Table 1**). Because the dosages for CBD and some ASMs are weight-based, the trial populations were categorized into 4 age groups (2-5, 6-11, 12-17, and 18-55 years) and analyzed to determine the mean weight and proportion of patients in these groups. Age groups were combined into one group for the analysis of QALY gains and costs to improve statistical power, using the mean age as the base case.

### Number of Seizures and Number of Seizure-Free Days

The number of seizures and seizure-free days included within the health states was determined using data from the RCTs. For LGS, the health states included 55 or fewer and more than 55 seizures per month, and the sub-health states were 15 or fewer and more than 15 seizure-free days per month (**Figure 1**). For DS, the health states were categorized as 12 or fewer and more than 12 seizures per month, and sub-health states included 18 or fewer and more than 18 seizure-free days per month (**Figure 1**). Model health state probabilities on seizure frequency and seizure-free days were based on PLD from the RCTs (for patients entering the OLE), as well as the OLE.^22–27^ PLD from the RCTs were used in Cycle 1 for the CBD-treated group and the placebo group.^22–25^ PLD data from the OLE were used in Cycles 2 to 9 for the CBD-treated group.^26,27^ Long-term cycles (Cycles 10+) for the CBD-treated group used the assumption that patients remain in the same health state following Cycle 9. This assumption is supported by the OLE and the US Expanded Access Program (EAP), in which CBD has shown sustained effectiveness over time (**Supplementary Figure S1**).^26,27,39,40^ For the placebo group, subsequent and long-term cycles (Cycles 2+) used the assumption that the placebo effect observed in Cycle 1 was maintained for the lifetime of the patient, in the absence of long-term data.

### Safety

Treatment-emergent adverse events (AEs) of special interest, reported in at least 3% of patients treated with CBD plus usual care and at least 1% of patients in the usual care arm, were included in the model. AE and serious AE (SAE) rates were based on events observed during the treatment period for the CBD and placebo arms of the RCTs.^22–25^ As AEs generally occur within the first few months following treatment initiation,^41,42^ it was assumed that AEs do not occur from Cycle 10 onwards. The model used the assumption that AEs may occur up to Cycle 9 (representing >2 years) at the same rate as observed in the first 14 weeks in the RCTs.^22–25^

### Discontinuation and Stopping Rule Rates

Discontinuation rates were applied only to patients in the CBD arm, given that patients do not discontinue from usual care (comparator arm) in clinical practice. The short-term discontinuation rates for Cycle 1 were derived from AEs experienced during the RCTs.^22–25^ The mid-term discontinuation rates for Cycles 2-9 were based on all-cause patient discontinuations observed during the OLE.^26,27^ The long-term discontinuation rates for Cycles 10 and above were assumed to be 4.17% based on a Kaplan-Meier analysis from the US-based EAP for CBD to account for the real-world persistence on treatment.^39,40^ Lastly, stopping rule discontinuation rates were based on the proportion of patients who did not achieve a clinically meaningful or desired level of seizure reduction during the OLE (≥30% reduction in seizure frequency following initiation of CBD).^26,27^ In the base case, the stopping rule was applied as a one-off conditional discontinuation rate at 6, 12, and 24 months. A scenario analysis with a long-term discontinuation rate of 10% applied in the seizure health states was conducted to account for any reduced treatment effect over time.

### Mortality

All-cause, age-dependent probabilities of death were derived from national life tables for the Netherlands.^43^ The additional risks associated with LGS- and DS-specific mortality were also considered. In the absence of specific SUDEP and non-SUDEP data for LGS, it was assumed in the model that SUDEP and non-SUDEP mortality rates for LGS were the same as the rates observed in DS.^11^ Although evidence suggests that the risk of death may be elevated in patients having more seizures,^11^ it was assumed in the base case model that mortality rates were equal across seizure frequency health states. An alternative scenario was conducted which assumed that patients with seizure freedom have a lower risk of mortality than patients with a higher number of seizures (0.71 risk ratio associated with seizure-free patients).

### Costs and Resource Use

Drug acquisition, disease management, AEs, and societal costs were included, with unit costs sourced from published literature using a 2019/2020 price year in euros (**Tables S2 and S3**; see **Online Supplementary Material**). As validated by clinical experts, treatment administration costs were not relevant as all routine ASMs are administered orally, and resource use or costs associated with routine patient monitoring were not considered because monitoring requirements are similar for CBD and ASMs considered to be part of usual care. Societal parameters comprised out-of-pocket expenses for both patients and caregivers, including travel costs associated with hospital visits and the time cost of family and professional care. Productivity losses were not considered in the analysis as Dutch clinical experts confirmed that most patients with LGS or DS are unemployed. Healthcare resource utilization (HCRU) data were collected via interviews with three UK clinical experts, with estimates validated by Dutch clinical experts (**Table 1** and **Tables S4-S5**).

### Utilities

In the pivotal RCTs for CBD, several exploratory QoL and patient/caregiver-reported outcome measures were included; however, many of these measures are not validated in LGS or DS and may not detect treatment effects in short-term clinical trial settings. Furthermore, it has been noted that the EuroQol 5-level questionnaire (EQ-5D-5L) is not suitable because it does not capture the variation of health-related QoL (HRQoL) in patients with severe epilepsy over time, as treatment-resistant patients may experience days with multiple seizures and other days with no seizures at all.^44,45^ Owing to the lack of HRQoL data in patients with LGS or DS when this study was conducted, HRQoL estimates were based on data on file from a vignette study conducted in the UK and Swedish general population, using the time trade-off method, and were adjusted to match the health states used in the economic model and then validated by Dutch clinical experts (**Table 1**; see **Online Supplementary Material** for methodology). The findings of zero and/or negative utilities (ie, a state worse than death) for the most severe health states were not unexpected given the extreme number of seizures and the consequent impact on the QoL of patients. As per the ZiN guidelines,^33^ the base case did not consider the QoL of caregivers; however, this was considered in scenario analyses because a key driver of the value of CBD is the potential positive impact on caregiver QoL due to a reduction in seizure frequency. A short-term (1 cycle) disutility for SAEs was applied. Disutilities associated with the relevant SAEs were based on a disutility value of −0.12 for ‘severe side-effects.’^46^

### Sensitivity Analyses

Parameter uncertainty was assessed via one-way sensitivity analysis (OWSA), which varied each parameter individually between the upper and lower bounds of confidence intervals within prespecified probabilistic distributions assigned to each parameter. Where the standard error was unavailable to calculate upper and lower confidence intervals, this was assumed to be ±20% of the mean value. A tornado diagram was developed to illustrate the level of uncertainty of the incremental cost-effectiveness ratio (ICER) based on the upper and lower bounds of the OWSA parameters.

The probabilistic sensitivity analysis (PSA) assigned distributions to the model parameters and ran 10 000 simulations to further explore parameter uncertainty. Mean incremental results were recorded and illustrated through an incremental cost-effectiveness plane and cost-effectiveness acceptability curve plot.

Scenario analyses were conducted to test uncertainty around structural and parametric assumptions. The alternative scenarios explored are shown in **Table 2**.

**Table 2.** Base Case and Key Scenario Analyses for Lennox-Gastaut Syndrome and Dravet Syndrome

	**Total Costs (€)**				**Total QALYs**				**ICER, €/QALY**	
	**LGS**		**DS**		**LGS**		**DS**		**LGS**	**DS**
	**CBD**	**Usual Care**	**CBD**	**Usual Care**	**CBD**	**Usual Care**	**CBD**	**Usual Care**		
**Base case**	**2 365 876**	**2 337 538**	**2 222 650**	**2 246 291**	**6.749**	**5.431**	**15.813**	**14.944**	**21 493**	**Dominating**
Applied a 2023 cost year	2 686 073	2 667 187	2 507 247	2 545 925	6.749	5.431	15.813	14.944	14 324	Dominating
Healthcare perspective (excludes replacement costs)	1 084 229	1 010 424	983 386	937 731	6.749	5.431	15.813	14.944	55 978	52570
Applied 12-17 y group weight to the 18-55 y group (DS only)	N/A	N/A	2 236 433	2 255 965	N/A	N/A	15.813	14.944	N/A	Dominating
Assume patients in both model arms remain in their health states following Cycle 1	2 381 055	2 337 538	2 216 519	2 246 291	6.627	5.431	15.903	14.944	36 380	Dominating
Alternative seizure thresholds^a,b^	2 341 096	2 309 790	2 205 285	2 220 720	5.259	4.282	16.187	15.511	32 051	Dominating
Relative treatment effect is not maintained	2 367 967	2 344 732	2 246 248	2 324 616	6.402	4.644	15.591	14.345	13 216	Dominating
Target population: starting age of 2 y	3 311 261	3 310 538	2 889 889	2 951 745	6.982	5.652	16.326	15.448	543	Dominating
Mean weight of adults assumed the same as the 12-17 y age group (DS only)	N/A	N/A	2 236 433	2 255 965	N/A	N/A	15.813	14.944	N/A	Dominating
Time horizon: 20 y	1 761 516	1 730 599	1 629 781	1 646 102	3.943	2.859	8.464	7.833	28 538	Dominating
Time horizon: 30 y	2 040 740	2 010 982	1 901 404	1 921 581	4.940	3.748	10.996	10.259	24 969	Dominating
No. of caregivers: 2	2 365 876	2 337 538	2 222 650	2 246 291	-15.438	−18.863	7.378	5.424	8272	Dominating
Utilities associated with non-drop seizures included (LGS only)	2 365 876	2 337 538	N/A	N/A	3.387	1.917	N/A	N/A	19 281	N/A
Utilities associated with non-convulsive seizures included (DS only)	N/A	N/A	2 222 650	2 246 291	N/A	N/A	13.430	12.312	N/A	Dominating
Mortality RR in seizure-free health states: 0.71	2 372 579	2 337 538	2 241 455	2 252 557	6.831	5.431	16.191	15.132	25 028	Dominating
Varied proportion for ICU admissions within hospitalizations: 50% in general ward vs 50% in ICU	2 380 252	2 353 686	2 247 975	2 275 288	6.749	5.431	15.813	14.944	20 150	Dominating
10% long-term discontinuation rate applied to seizure health states	2 345 962	2 337 538	2 211 322	2 246 291	6.526	5.431	15.748	14.944	7693	Dominating
Lag discontinuation applied for 6 months	2 372 464	2 337 538	2 228 066	2 246 291	6.749	5.431	15.813	14.944	26 490	Dominating
Varied AE disutilities: doubling	2 365 876	2 337 538	2 222 650	2 246 291	6.749	5.431	15.811	14.944	21 500	Dominating
Varied AE disutilities: tripling	2 365 876	2 337 538	2 222 650	2 246 291	6.749	5.431	15.810	14.944	21 506	Dominating
Varied AE costs: doubling	2 365 985	2 337 582	2 222 838	2 246 390	6.749	5.431	15.813	14.944	21 543	Dominating
Varied AE costs: tripling	2 366 095	2 337 627	2 223 027	2 246 489	6.749	5.431	15.813	14.944	21 592	Dominating
No. of cycles for which AE disutilities were applied: 9 cycles	2 365 876	2 337 538	2 222 650	2 246 291	6.748	5.431	15.807	14.944	21 523	Dominating
Utility source: DS utilities (time trade-off values)	2 365 876	2 337 538	N/A	N/A	9.418	8.231	N/A	N/A	23 867	N/A
Utility source: LGS utilities (time trade-off values)	N/A	N/A	2 222 650	2 246 291	N/A	N/A	10.849	9.484	N/A	Dominating
Applied resource use estimates from the literature	2 406 702	2 376 848	2 212 870	2 232 014	6.749	5.431	15.813	14.944	22 643	Dominating
Applied same hours of care for seizure health states	2 326 838	2 287 990	2 185 668	2 199 483	6.749	5.431	15.813	14.944	29 465	Dominating
Applied same percentage of patients institutionalized for seizure-free and least severe seizure health states	2 183 066	2 159 546	2 079 448	2 092 754	6.749	5.431	15.813	14.944	17 839	Dominating

Abbreviations: AE, treatment-emergent adverse event; CBD, cannabidiol; DS, Dravet syndrome; ICER, incremental cost-effectiveness ratio; ICU, intensive care unit; LGS, Lennox-Gastaut syndrome; N/A, not applicable; QALY, quality-adjusted life year; RR, risk ratio.

^a^Alternative seizure thresholds for LGS include the following health states: seizure free, ≤84 seizures, and >84 seizures (days with seizures); ≤12 days and >12 days (seizure-free days).

^b^Alternative seizure thresholds for DS include the following health states: seizure free, ≤25 seizures, and >25 seizures (days with seizures); ≤15 days and >15 days (seizure-free days).

### Additional Model Outputs

The total number of emergency department visits, nurse visits, hospitalizations, and institutionalizations over a patient’s lifetime were calculated using model data, by multiplying the number of patients within each health state over the model time horizon by the probability of each visit, for each treatment arm.

## RESULTS

### Base Case

In the base case analysis over a lifetime horizon, the total costs for patients with LGS were €2 365 876 for CBD and €2 337 538 for usual care. The total QALYs were 6.749 for CBD and 5.431 for usual care in patients with LGS. Therefore, CBD plus usual care led to additional costs of €28 338 and increased QALYs of 1.318 compared with usual care alone in patients with LGS. The resulting ICER was €21 493/QALY in patients with LGS. In patients with DS, the total costs were €2 222 650 for CBD and €2 246 291 for usual care. The total QALYs were 15.813 for CBD and 14.944 for usual care in patients with DS. CBD plus usual care dominated usual care alone, with cost savings of €23 642 and increased QALYs of 0.868. The ICERs identified for CBD plus usual care in LGS and DS were both below the WTP threshold of €80 000/QALY in the Netherlands for these conditions.

### One-Way Sensitivity Analysis

**Figure 2.**
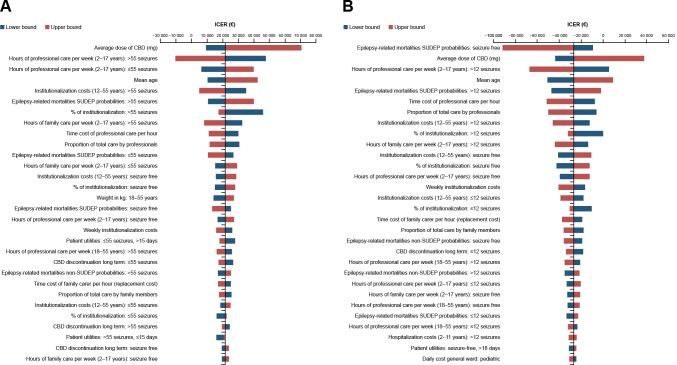
Tornado Diagrams (Incremental Cost-Effectiveness Ratio) Abbreviations: CBD, cannabidiol; DS, Dravet syndrome; ICER, incremental cost-effectiveness ratio; LGS, Lennox-Gastaut syndrome; SUDEP, sudden unexpected death in epilepsy. Tornado diagram of the one-way sensitivity analysis for CBD plus usual care vs usual care alone in LGS (**A**) and DS (**B**) (ICER).

**Figure 3.**
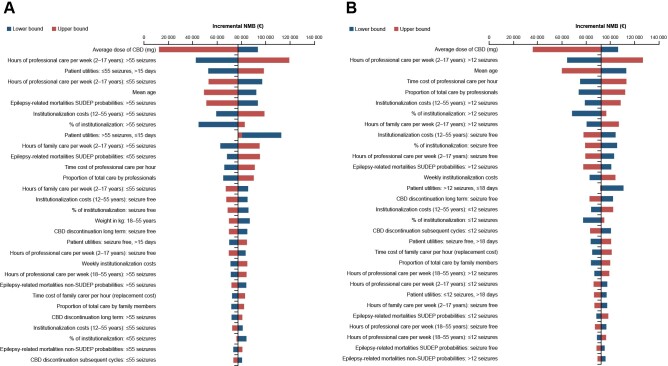
Incremental Net Monetary Benefit Abbreviations: CBD, cannabidiol; DS, Dravet syndrome; ICER, incremental cost-effectiveness ratio; LGS, Lennox-Gastaut syndrome; NMB, net monetary benefit; SUDEP, sudden unexpected death in epilepsy. Incremental net monetary benefit for CBD plus usual care vs usual care alone in LGS (**A**) and DS (**B**).

The results of the OWSA for both indications demonstrated that the model is robust to sensitivity and key scenario analyses (**Figure 2**). For both LGS and DS, the ICER was most sensitive to the average dose of CBD, with a range of €9226 to €70 561 for patients with LGS and dominating to €37 584 for DS. Other key drivers of the model included the number of hours of professional care per week and the mean age of patients. The incremental net monetary benefit for CBD plus usual care vs usual care alone associated with LGS and DS was positive (**Figure 3**).

### Probabilistic Sensitivity Analysis

**Figure 4.**
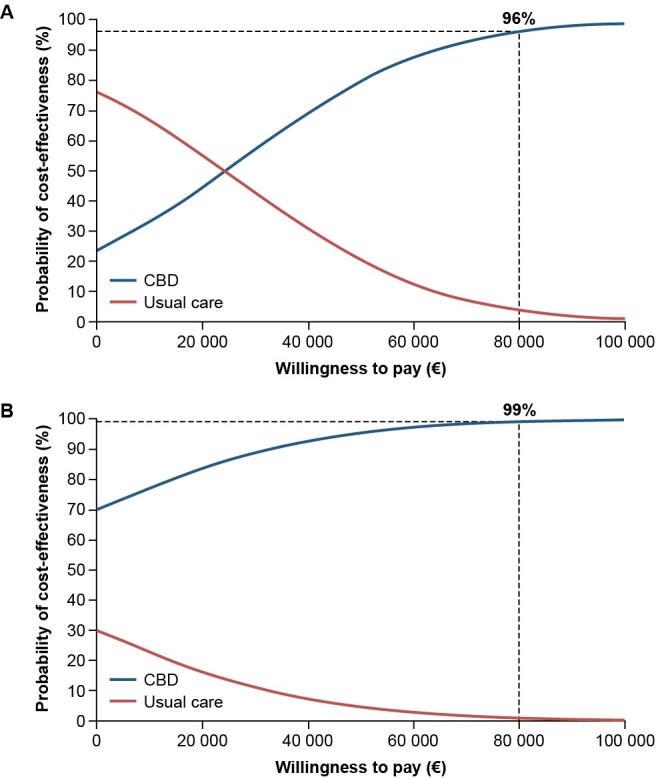
Cost-Effectiveness Acceptability Curves Abbreviations: CBD, cannabidiol; DS, Dravet syndrome; LGS, Lennox-Gastaut syndrome. Probabilistic sensitivity analysis cost-effectiveness acceptability curves in patients with LGS (**A**) and DS (**B**).

The results of the PSA, at a WTP threshold of €80 000/QALY for each condition, showed that the probability of CBD plus usual care being cost-effective compared with usual care alone was 96% for patients with LGS and 99% for patients with DS (**Figure 4**).

### Scenario Analysis

Scenario analysis results are shown in **Table 2**. For patients with LGS, the most influential scenario on the ICER was the application of alternative seizure thresholds. Additional key influential scenarios included an updated price year of 2023, the time horizon, mortality risk ratio applied to the seizure-free health state, and hours of care. For DS, CBD dominated usual care alone across all scenario analyses.

### Additional Model Outputs

Additional analysis outputs demonstrated that over a lifetime horizon, patients with LGS or DS taking CBD plus usual care had a reduction of 66 and 103 emergency department visits, 150 and 147 nurse visits, 85 and 94 hospitalizations, and 125 and 112 institutionalizations, respectively, compared with usual care alone (**Supplementary Tables S6 and S7**). The total average time spent in hospital over a lifetime in patients with LGS and DS taking CBD plus usual care was 9 and 13 days, respectively, compared with 179 and 201 days in patients taking usual care alone, respectively.

## DISCUSSION

This analysis aimed to provide evidence for the cost-effectiveness of CBD plus usual care to aid treatment decision making for patients with LGS or DS in the Netherlands. CBD (Epidyolex®) demonstrated efficacy in reducing seizure frequency in patients with LGS or DS compared with placebo in the pivotal RCTs^22–25^ and has shown effectiveness in increasing the number of seizure-free days in patients with LGS and DS in a post-hoc analysis and EAP data.^38,47^ A sustained response to CBD treatment has also been demonstrated in an OLE in patients with these types of refractory epilepsies.^26,27^ Along with these findings, it has been reported that reduced seizure frequency and increased number of seizure-free days may be associated with improved patient and caregiver QoL.^8,20,38^ Based on these findings, the model described here assumed that a decrease in seizure frequency and an increase in seizure-free days would result in cost savings and improved HRQoL outcomes for patients with LGS or DS.

Using a Markov state-transition cohort model and a WTP threshold of €80 000 for each condition in the Netherlands, the base case cost-utility analysis demonstrated the cost-effectiveness of an average of 12 mg/kg/day CBD plus usual care in patients with treatment-refractory LGS or DS aged 2 years or older in the Netherlands.

Despite the higher treatment costs associated with CBD plus usual care compared with usual care alone, the cost-effectiveness of add-on CBD was demonstrated in the present study owing to the decreased health state and societal costs, and increased patient QoL following treatment with add-on CBD. For example, based on the input data from the model applying a 90-year time horizon, lifetime HCRU in terms of emergency department and nurse visits, hospitalizations, average time spent in hospital, and institutionalizations was lower with CBD plus usual care compared with usual care alone.

Over the model time horizon, there were fewer nurse visits in a hospital for CBD plus usual care vs usual care alone, resulting in lower travel costs. Over this period, there were also fewer hours of care required for patients receiving CBD plus usual care, resulting in lower caregiver costs (both formal and informal caregivers) vs usual care alone. In a cohort of patients with treatment-refractory epilepsy, CBD treatment has previously been reported to decrease seizure-related hospital admissions,^48^ which has been linked with improved patient and caregiver QoL stemming from less disruption to family life.^8^

The sensitivity analyses performed here showed the results of the base case analysis were robust. All modeled scenarios demonstrated ICERs less than the WTP threshold of €80 000/QALY for LGS and DS in the Netherlands, including those using an updated price year of 2023 to account for any effects on healthcare system costs following the COVID-19 pandemic. In addition, the results of applying a scenario analysis from a healthcare perspective—to remove the impact of excluding replacement costs—were still below the €80 000/QALY WTP threshold. The results of the OWSA demonstrated that the ICER is most sensitive to the average dose of CBD for both LGS and DS, and the hours of professional care, health state utilities, and mean age. The positive incremental net monetary benefit in favor of CBD plus usual care for LGS and DS implies that the additional value of CBD plus usual care is greater than the extra cost compared with usual care alone. In the model used in the present study, at the WTP threshold for each condition, the results from the PSA determined that the probability of add-on CBD being cost-effective was 96% in patients with LGS and 99% in patients with DS.

One of the key strengths of this analysis is the incorporation of clinical evidence from the phase 3 RCTs, which are the largest clinical trials conducted in patients with LGS or DS to date. The RCTs for CBD included a number of exploratory QoL and patient/caregiver-reported outcome measures^22–25^; however, these instruments were designed for use in all epilepsies and are not validated in patients with LGS or DS. Owing to the severe refractory nature of LGS and DS, the QoL questions in these instruments can be challenging or not applicable when applied to these patients, leading to missing data and nonmeaningful conclusions. Furthermore, because clinical trials are relatively short compared with the lifetime of treatment and potential for beyond-seizure benefits, QoL outcomes can be variable between studies. To overcome the challenges of collecting robust utility data from patients with LGS or DS, our model used data from a time trade-off vignette study. Another strength of the present analysis is that this model used HCRU inputs from patients, carers, and clinical experts that have been reviewed and validated by clinical experts in multiple countries, including the Netherlands. In addition, estimates from the literature were applied as scenarios to validate the base case model assumptions. Lastly, to capture the effects of absolute seizure reductions and changes in seizure-free days on patients and caregivers, the model structure was also developed to incorporate the wider benefits of CBD treatment on society.

There are various limitations to this study pertaining to the collection of data and the assumptions of the model. First, long-term clinical data for CBD relative to the 90-year duration of the analysis are lacking, so the model assumed that patients receiving CBD continue to stay in the same health state (after Cycle 9) for the remaining duration of the analysis. It should be noted that this is an issue for all emerging treatments where there is a need to extrapolate trial data over long time horizons for modeling purposes. Given that CBD has demonstrated durable efficacy in the OLE^26,27^ and an EAP,^39,40^ the model assumed that this durability would be translated to a lifetime. It should be noted that the NICE committee recommended that economic models include the diminishing effectiveness of CBD and not to assume that CBD prolongs life.^29,30^ Based on this recommendation, a scenario of diminishing CBD effectiveness was analyzed in this study; a lag discontinuation scenario was conducted which assumes that patients with a loss of CBD effect continue treatment for an extra 2 cycles and incur the extra cost with no treatment benefit.

In addition, owing to the lack of data on SUDEP in LGS, a further limitation is the assumption that SUDEP has a similar incidence in LGS and DS. Despite there being some supporting evidence, further studies are required to determine the risk of SUDEP among patients with LGS, and also among patients with DS with different seizure severities.^11^ Although it would have been ideal to use data for all model parameters from real patient data,^49^ data are lacking on HCRU, utilities, and patient/caregiver QoL in patients with LGS or DS. As such, we used elicitation methods for this model, which were validated by Dutch clinical experts. The HCRU data used in the model were collected by interviews of 3 UK clinical experts, which may have led to bias and uncertainty. Similarly, the utilities and QoL data used in the model were collected by vignettes using time trade-off methods in the Swedish and UK population. Although these vignette-based methods are useful when published data are limited, they can also lead to bias and are limited by the health state descriptions that may not include all aspects of a patient’s experience.^50^

A final limitation is that while previous studies have shown the significant impact of LGS and DS on family and caregiver QoL, including the ability to work,^8^ the present model did not consider this, based on the ZiN guidelines.^33^ To account for this and to align with guidance from the NICE committee,^29,30^ we included the impact of caregiver QoL in the scenario analyses by adjusting the utility decrements.

## CONCLUSIONS

Based on a WTP threshold of €80 000 for each condition, and a PSA cost-effectiveness probability of 96% in LGS and 99% in DS, this analysis showed that treatment with CBD plus usual care is cost-effective compared with usual care alone in patients with treatment-resistant LGS or DS in the Netherlands. These findings were robust to sensitivity and scenario analyses, validating the chosen model parameters and assumptions.

### Disclosures

J.S. was employed by FIECON at the time of the study and received grant/research support from Jazz Pharmaceuticals, Inc.; J.S. is now an employee of Jazz Pharmaceuticals UK Ltd. and holds stock and/or stock options in Jazz Pharmaceuticals, plc. S.B. is an employee of Jazz Pharmaceuticals UK Ltd. and holds stock and/or stock options in Jazz Pharmaceuticals, plc.

### Data and Material Availability

All relevant data are provided with the manuscript and supporting files.

### Ethics Approval

The present study was based on clinical trial data from GWPCARE1 (NCT02091206, NCT02091375), GWPCARE2 (NCT02224703), GWPCARE3 (NCT02224560), GWPCARE4 (NCT02224690), and the corresponding OLE GWPCARE5 (NCT02224573). The trials were approved by an institutional review board or ethics committee at each participating site and were conducted in accordance with the principles of the Declaration of Helsinki and the International Conference on Harmonisation Tripartite Guideline on Good Clinical Practice. GWPCARE1 was conducted in the UK and the US; GWPCARE2 was conducted in Australia, Israel, the Netherlands, Poland, Spain, and the US; GWPCARE3 was conducted in France, Spain, the UK, and the US; GWPCARE4 was conducted in the Netherlands, Poland, and the US; and GWPCARE5 was conducted in France, Israel, the Netherlands, Poland Spain, the UK, and the US.

### Informed Consent

All patients or their caregivers provided written informed consent, and patients developmentally mature enough to understand the trial provided assent.

### Code Availability

The cost-effectiveness model used in the study is available from the corresponding author on reasonable request.

## Supplementary Material

Online Supplementary Material
