# Supplementary material for: Cost-Utility Analysis of Add-on Cannabidiol vs Usual Care Alone for the Treatment of Seizures in Patients With Treatment-Resistant Lennox-Gastaut Syndrome or Dravet Syndrome in the Netherlands
Source: J Health Econ Outcomes Res. 2024 Dec 23;11(2):168–79. doi: 10.36469/001c.126071 (PMC11686597; doi:10.36469/001c.126071)
Supplement: Online Supplementary Material [file jheor_2024_11_2_126071_259637.pdf]

Journal of Health Economics and Outcomes Research

Online Supplementary Material

Cost-Utility Analysis of Add-on Cannabidiol vs Usual Care Alone for the Treatment of Seizures in Patients With Treatment-Resistant Lennox-Gastaut Syndrome or Dravet Syndrome in the Netherlands. *JHEOR*. 2024;11(2):168-179. doi:10.36469/jheor.2024.126071

Table of Abbreviations

Figure S1: Reduction in Drop (LGS) or Convulsive (DS) Seizure Frequency from Baseline During GWPCARE5

Table S1: Underlying Assumptions and Justifications for the Markov Model

Table S2: Discounted and Undiscounted Incremental and Total Costs for LGS

Table S3: Discounted and Undiscounted Incremental and Total Costs for DS

Table S4: Resource Costs and Annual Resource Use Associated With Each Health State for Patients With LGS

Table S5: Resource Costs and Annual Resource Use Associated With Each Health State for Patients With DS

Table S6: Model Predictions of Total Number and Costs per Visit for ED Visits, Nurse Visits, Hospitalizations, and Institutionalizations in Patients With LGS

Table S7: Model Predictions of Total Number and Costs per Visit for ED Visits, Nurse Visits, Hospitalizations, and Institutionalizations in Patients With DS

Table S8: Vignettes and the LGS Economic Model Structure

Table S9: Vignettes and the DS Economic Model Structure

This supplementary material has been provided by the authors to give readers additional information about their work.

This is an open-access article distributed under the terms of the Creative Commons Attribution 4.0 International License (CCBY-4.0). View this license's legal deed at http://creativecommons.org/licenses/by/4.0 and legal code at http://creativecommons.org/licenses/by/4.0/legalcode for more information.

Table of Abbreviations	
AE	Treatment-emergent adverse event
ASM	Antiseizure medication
CBD	Cannabidiol
DS	Dravet syndrome
EAP	Expanded Access Program
EEG	Electroencephalogram
ED	Emergency department
EMA	European Medicines Agency
EQ-5D-5L	EuroQol 5-level questionnaire
HCRU	Healthcare resource utilization
HRQoL	Health-related quality of life
ICER	Incremental cost-effectiveness ratio
ICU	Intensive care unit
LGS	Lennox-Gastaut syndrome
N/A	Not applicable
NHS	National Health Service
NICE	National Institute for Health and Care Excellence
NMB	Net monetary benefit
OLE	Open-label extension
OWSA	One-way sensitivity analysis
PLD	Patient-level data
PSA	Probabilistic sensitivity analysis
RCT	Randomized controlled trial
RR	Risk ratio
QALY	Quality-adjusted life year
QoL	Quality of life
SAE	Serious adverse event
SE	Standard error
SMC	Scottish Medicines Consortium
SUDEP	Sudden unexpected death in epilepsy
TTO	Time trade-off
VAS	Visual analog scale
WTP	Willingness-to-pay
ZiN	Zorginstituut Nederland

Figure S1. Reduction in Drop (LGS) or Convulsive (DS) Seizure Frequency from Baseline During GWPCARE5

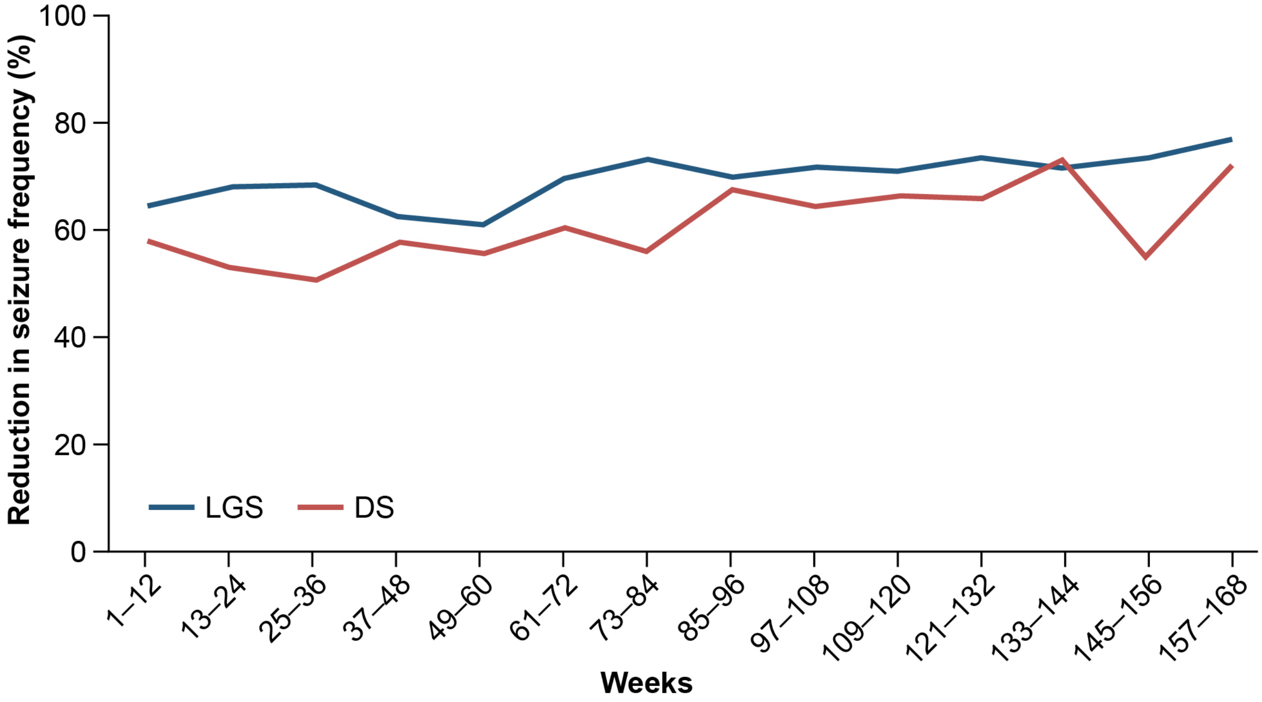

Table S1. Underlying Assumptions and Justifications for the Markov Model

Assumption	Justification
**Time horizon**	
A maximum lifetime horizon of 90 years is used.	A lifetime horizon was selected for the base case as this analysis period was considered to be sufficiently long enough for most patients to discontinue their therapy, and to take into account all relevant costs and outcomes associated with LGS and DS.
**Model structure**	
Markov cohort model structure was used instead of a micro-simulation.	Analysis of the patient-level data from the pivotal phase 3 trials for LGS and DS showed that the treatment effect was not significantly different across the patient subgroups stratified by age, sex, number of ASMs previously taken, and use of specific ASMs. Furthermore, all cost-effectiveness analyses conducted for LGS and DS to date have also been based on the Markov cohort model structure. The model structure (including the health state distributions) was validated by a Dutch clinical expert.
When patients discontinue CBD, they are assumed to follow the disease severity distribution as observed in the usual care arm at the end of Cycle 1.	This assumption was made because there are no data on seizure outcomes following withdrawal of CBD.
The distribution of patients in the different health states at the end of Cycle 1 in the comparator arm was applied for the duration of the analysis (ie, without re-transitioning): ie, patients were assumed to remain in their final health state at the end of Cycle 1 for the rest of the time horizon.	This was done because there are no longitudinal natural history data available in these very rare conditions to estimate transitions beyond 3 months on usual care. Therefore, this 'snapshot' of health states in the placebo arm at the end of the GWPCARE trials represents the best data available to predict the natural history of disease progression among patients not treated with CBD.
**Clinical effectiveness**	
In the model, patients experiencing seizures were assumed to be at risk of death from SUDEP and non-SUDEP causes (such as status epilepticus, drowning, and asphyxia).<br>It was assumed that the risk of SUDEP and non-SUDEP were the same for all health states where patients continue to experience seizures, and a lower risk was assumed only for the seizure-free health state (as a scenario analysis).	Conservative assumption
It has been assumed that AEs occur during Cycles 1-9. In our analyses, only the costs associated with managing AEs were considered.<br>In addition to the management costs, disutilities associated with SAEs were also considered in the model and were assumed to affect patients only for a short period of time (ie, 1 cycle).	AEs generally occur within the first few months following treatment initiation. Once a patient is stable on CBD or any of the conventional ASMs, the incidence of AEs is expected to be very low.
We have assumed a 0.5% discontinuation rate for the seizure-free health state from Cycle 10 onwards ('long-term') as a conservative estimate.	Having no discontinuations in patients who are seizure free in the long term is unlikely to be fully representative of a real-world clinical setting. Therefore, a 0.5% discontinuation rate per long-term cycle was assumed, reflecting that long-term persistence on any treatment is unlikely to be 100% in a chronic condition. This was validated by a Dutch clinical expert.
We have assumed that patients under the age of 18 do not work, and that all patients with LGS or DS are unable to work.	This assumption was made to reflect current practice in the Netherlands, and was validated by a Dutch clinical expert.
We have assumed that all patients in the model are dead once they reach the age of 100.	Common modeling practice

Table S2. Discounted and Undiscounted Incremental and Total Costs for LGS

Cost Categories, €	CBD 12 mg/kg/day	Usual Care	Difference
Discount rate 4%			
Treatment cost	144 017	46977	97 041
Health state cost	940 102	963 403	−23 301
AE cost	110	45	65
Societal cost	1 281 646	1 327 113	−45 467
**Total cost**	**2 365 876**	**2 337 538**	**28 338**
Undiscounted			
Treatment cost	246 900	113 266	133 634
Health state cost	2 485 410	2 531 114	−45 704
AE cost	112	46	67
Societal cost	2 190 613	2 252 515	−61 901
**Total cost**	**4 923 036**	**4 896 940**	**26 096**

Note: Based on a 2019/2020 price year.

Table S3. Discounted and Undiscounted Incremental and Total Costs for DS

Cost Categories, €	CBD 12 mg/kg/day	Usual care	Difference
Discount rate 4%			
Treatment cost	207 450	123 028	84 422
Health state cost	775 746	814 604	−38 857
AE cost	189	99	90
Societal cost	1 239 264	1 308 560	−69 296
**Total cost**	**2 222 650**	**2 246 291**	**−23 642**
Undiscounted			
Treatment cost	437 281	302 147	135 133
Health state cost	2 249 526	2 334 301	−84 775
AE cost	193	102	91
Societal cost	2 065 870	2 166 857	−100 986
**Total cost**	**4 752 869**	**4 803 406**	**−50 537**

Note: Based on a 2019/2020 price year.

Table S4. Resource Costs and Annual Resource Use Associated With Each Health State for Patients With LGS

Resource and Unit Cost	Health State	Age Group of Patients With LGS			
		**2-17 Years**	**SE**	**18-55 Years**	**SE**
**Nurse visit,** No. per year (€34.66 per visit)	Seizure-free	2.0	0.4	2.0	0.4
	≤55 seizures	4.0	0.8	4.0	0.8
	>55 seizures	4.0	0.8	4.0	0.8
**Rehabilitation physician visit,** No. per year (€68.28 per visit)	Seizure-free	2.0	0.4	1.0	0.2
	≤55 seizures	2.0	0.4	1.0	0.2
	>55 seizures	2.0	0.4	1.0	0.2
**Pediatric epileptologist (<18 years)/neurologist (≥18 years) visit,** No. per year (€106.09 [2-17 years] per visit/€103.99 [18-55 years] per visit)	Seizure-free	1.0	0.2	0.5	0.1
	≤55 seizures	2.0	0.4	1.0	0.2
	>55 seizures	4.8	1.0	1.9	0.4
**Pediatrician visit,** No. per year (€106.09 [2-17 years] per visit)	Seizure-free	2.0	0.4	0.0	0.0
	≤55 seizures	4.0	0.8	0.0	0.0
	>55 seizures	9.5	1.9	0.0	0.0
**Emergency department visit,** No. per year (€272.05 per visit)	Seizure-free	0.0	0.0	0.0	0.0
	≤55 seizures	2.0	0.4	1.0	0.2
	>55 seizures	4.0	0.8	2.0	0.4
**Phone call follow-up with epileptologist (<18 years)/ neurologist (≥18 years),** No. per year (€18.16 per call)	Seizure-free	0.0	0.0	0.0	0.0
	≤55 seizures	2.0	0.4	1.0	0.2
	>55 seizures	7.7	1.5	3.8	0.8
**Dentist visits,** No. per year (€22.91 per visit)	Seizure-free	2.0	0.4	2.0	0.4
	≤55 seizures	2.0	0.4	2.0	0.4
	>55 seizures	2.0	0.4	2.0	0.4
**Hospitalizations,**[a] No. per year (€658.60 [2-17 years]/€499.99 [18-55 years] per general ward visit; €1245.77 per ICU visit)	Seizure-free	0.0	0.0	0.0	0.0
	≤55 seizures	0.5	0.1	1.5	0.3
	>55 seizures	2.8	0.6	2.8	0.6
**Institutionalization,**[b] % of patients (€1536.74 [18-55 years only] per weekly entry)	Seizure-free	0	0.0	50	10.0
	≤55 seizures	0	0.0	90	18.0
	>55 seizures	0	0.0	90	18.0
**Rescue medication intake,** No. of times per year (€0.17 per use)	Seizure free	0.0	0.0	0.0	0.0
	≤55 seizures	2.0	0.4	2.0	0.4
	>55 seizures	6.2	1.2	6.2	1.2
**Death** (0 for SUDEP; €1517.82 for non-SUDEP [1 ED visit + 1 day in ICU])	SUDEP	None		None	
	Non-SUDEP	1 visit to the ED and 10 days in ICU		1 visit to the ED and 5 days in ICU	
**AEs** (€34.66 [1 visit to specialist nurse] per event)	Aggression	1 visit to a specialized nurse			
	Decreased appetite	1 visit to a specialized nurse			
	Diarrhea	1 visit to a specialized nurse			
	Fatigue	1 visit to a specialized nurse			
	Irritability	1 visit to a specialized nurse			
	Lethargy	1 visit to a specialized nurse			
	Sedation	1 visit to a specialized nurse			
	Somnolence	1 visit to a specialized nurse			
	Rash	1 visit to a specialized nurse			

All data are based on clinical expert opinion gathered at a virtual meeting held on July 14, 2021, with 4 practicing clinicians (3 pediatric neurologists and 1 neurologist) from the Netherlands.
[a]Hospitalization: according to key opinion leaders interviewed, 95% of the patients hospitalized will be admitted to a general ward; the rest (5%) will go to the ICU.
[b]Only patients over 18 years of age are assumed to be institutionalized.

Table S5. Resource Costs and Annual Resource Use Associated With Each Health State for Patients With DS

Resource and Unit Cost	Health State	Age Group of Patients With DS			
		**2-17 years**	**SE**	**18-55 years**	**SE**
**Nurse visit**, No. per year (€34.66 per visit)	Seizure-free	2.0	0.4	2.0	0.4
	≤12 seizures	4.0	0.8	4.0	0.8
	>12 seizures	4.0	0.8	4.0	0.8
**Rehabilitation physician visit**, No. per year (€68.28 per visit)	Seizure-free	2.0	0.4	1.0	0.2
	≤12 seizures	2.0	0.4	1.0	0.2
	>12 seizures	2.0	0.4	1.0	0.2
**Pediatric epileptologist** (<18 years)/neurologist (≥18 years) visit, No. per year (€106.09 [2-17 years] per visit/€103.99 [18-55 years] per visit)	Seizure-free	1.0	0.2	0.5	0.1
	≤12 seizures	2.0	0.4	0.5	0.1
	>12 seizures	5.1	1.0	1.9	0.4
**Pediatrician visit**, No. per year (€106.09 [2-17 years] per visit)	Seizure-free	2.0	0.4	0.0	0.0
	≤12 seizures	4.0	0.8	0.0	0.0
	>12 seizures	10.2	2.0	0.0	0.0
**Emergency department visit**, No. per year (€272.05 per visit)	Seizure-free	0.0	0.0	0.0	0.0
	≤12 seizures	4.0	0.8	2.0	0.4
	>12 seizures	6.0	1.2	3.0	0.6
**Phone call follow-up with epileptologist** (<18 years)/neurologist (≥18 years), No. per year (€18.16 per call)	Seizure-free	0.0	0.0	0.0	0.0
	≤12 seizures	2.0	0.4	1.0	0.2
	>12 seizures	9.3	1.9	4.4	0.9
**Dentist visits**, No. per year (€22.91 per visit)	Seizure-free	2.0	0.4	2.0	0.4
	≤12 seizures	2.0	0.4	2.0	0.4
	>12 seizures	2.0	0.4	2.0	0.4
**Hospitalizations,**[a] No. per year (€658.60 [2-17 years] / €499.99 [18-55 years] per general ward visit; €1245.77 per ICU visit)	Seizure-free	0.0	0.0	0.0	0.0
	≤12 seizures	3.0	0.6	1.5	0.3
	>12 seizures	5.3	1.1	3.1	0.6
**Institutionalization,**[b] % of patients (€1536.74 [18-55 years only] per weekly entry)	Seizure-free	0	0.0	50	10.0
	≤12 seizures	0	0.0	90	18.0
	>12 seizures	0	0.0	90	18.0
**Rescue medication intake,** No. of times per year (€0.17 per use)	Seizure-free	0.0	0.0	0.0	0.0
	≤12 seizures	12.0	2.4	6.0	1.2
	>12 seizures	37.2	7.4	18.6	3.7
**Death** (0 for SUDEP; €1517.82 for non-SUDEP [1 ED visit + 1 day in ICU])	SUDEP	None		None	
	Non-SUDEP	1 visit to the ED and 10 days in ICU		1 visit to the ED and 5 days in ICU	
**AEs** (€34.66 [1 visit to specialist nurse] per event)	Aggression	1 visit to a specialized nurse			
	Decreased appetite	1 visit to a specialized nurse			
	Diarrhea	1 visit to a specialized nurse			
	Fatigue	1 visit to a specialized nurse			
	Irritability	1 visit to a specialized nurse			
	Lethargy	1 visit to a specialized nurse			
	Sedation	1 visit to a specialized nurse			
	Somnolence	1 visit to a specialized nurse			
	Rash	1 visit to a specialized nurse			

All data are based on clinical expert opinion gathered at a virtual meeting held on July 14, 2021, with 4 practicing clinicians (3 pediatric neurologists and 1 neurologist) from the Netherlands.
[a]Hospitalization: According to key opinion leaders interviewed, 95% of the patients hospitalized will be admitted to a general ward; the rest (5%) will go to the ICU.
[b]Only patients over 18 years of age are assumed to be institutionalized.

Table S6. Model Predictions of Total Number and Costs per Visit for ED Visits, Nurse Visits, Hospitalizations, and Institutionalizations in Patients With LGS

	CBD	Usual Care	Increment CBD vs Usual Care	Absolute Increment CBD vs Usual Care	% Absolute Increment CBD vs Usual Care
**Total No. of ED visits (€272.05 per visit)**					
Seizure-free	0.00	0.00	0	0	0
≤55 seizures	3.97	20.41	-16	16	25
>55 seizures	2.71	51.84	-49	49	75
Total	6.68	72.25	-66	66	100
**Total No. of nurse visits (€34.66 per visit)**					
Seizure-free	3.17	0.00	3	3	2
≤55 seizures	9.79	73.22	-63	63	42
>55 seizures	3.14	92.85	-90	90	60
Total	16.09	166.07	-150	150	100
**Total No. of hospitalizations (€658.60 [2-17 years]/€499.99 [18-55 years] per visit)[a]**					
Seizure-free	0.00	0.00	0	0	0
≤55 seizures	2.15	25.35	-23	23	27
>55 seizures	2.17	64.24	-62	62	73
Total	4.32	89.59	-85	85	100
**Total No. of institutionalizations (€1536.74 [18-55 years only] per weekly entry)**					
Seizure-free	2.57	0.00	3	3	2
≤55 seizures	3.32	58.30	-55	55	44
>55 seizures	0.76	73.83	-73	73	58
Total	6.65	132.13	-125	125	100

Costs shown represent those for 2-7 and 18-55 year age categories unless stated otherwise. Costs were derived using the Dutch costing tool.[1]
[a]Hospitalizations were assumed to be 95% to general wards and 5% to an intensive care unit (ICU). Costs for the ICU were €1245.77 per visit for all patients.

Table S7. Model Predictions of Total Number and Costs per Visit for ED Visits, Nurse Visits, Hospitalizations, and Institutionalizations in Patients With DS

	CBD	Usual Care	Increment CBD vs Usual Care	Absolute Increment CBD vs Usual Care	% Absolute Increment CBD vs Usual Care
**Total No. of ED visits (€272.05 per visit)**					
Seizure-free	0.00	0.00	0	0	0
≤12 seizures	5.82	45.08	−39	39	38
>12 seizures	2.54	66.66	−64	64	62
Total	8.36	111.74	−103	103	100
**Total No. of nurse visits (€34.66 per visit)**					
Seizure-free	7.00	6.03	1	1	1
≤12 seizures	6.66	78.56	−72	72	49
>12 seizures	1.84	77.44	−76	76	52
Total	15.50	162.04	−147	147	100
**Total No. of hospitalizations (€658.60 [2-17 years]/€499.99 [18-55 years] per visit)[a]**					
Seizure-free	0.00	0.00	0	0	0
≤12 seizures	4.36	33.81	−29	29	31
>12 seizures	2.28	66.45	−64	64	69
Total	6.65	100.25	−94	94	100
**Total No. of institutionalizations (€1536.74 [18-55 years only] per weekly entry)**					
Seizure-free	5.50	0.00	6	6	5
≤12 seizures	1.52	60.27	−59	59	52
>12 seizures	0.26	59.41	−59	59	53
Total	7.29	119.68	−112	112	100

Costs shown represent those for 2-17 and 18-55 year age categories unless stated otherwise. Costs were derived using the Dutch costing tool.[1]
[a]Hospitalizations were assumed to be 95% to general wards and 5% to an intensive care unit (ICU). Costs for the ICU were €1245.77 per visit for all patients.

UK AND SWEDISH VIGNETTE STUDY

Objective: To estimate health state utilities for states describing the burden of living with Lennox-Gastaut syndrome (LGS) or Dravet syndrome (DS) and caring for a child with LGS or DS.

Study Design: Part 1 consisted of developing health state vignettes describing the impact of seizure frequency and the number of seizure-free days per month on the quality of life of patients with LGS or DS and informal caregivers/parents of a child with LGS or DS. **Part 2** consisted of conducting time trade-off (TTO) interviews with members of the general public, using the developed health state vignettes to generate utilities for each health state.

Part 1: Development of the Health State Vignettes

Six health state vignettes were developed for each of the following patient and caregiver groups to describe the experience of living with LGS, living with DS, caring for a child with LGS, or caring for a child with DS. This resulted in a total of 24 health states, which were described in 12 LGS vignettes and 12 DS vignettes. The vignettes described the experience of caregivers or patients with a certain level of seizure frequency and number of seizure-free days per month; these health states in LGS and DS are shown in **Table S8** and **Table S9**, respectively. Vignettes were not developed for all possible health states to avoid excessive respondent burden, as a total of 18 and 16 health states are defined in the economic models for LGS and DS respectively. The study estimated 6 out of 9 patient/caregiver health states in the model for LGS and 6 out of 8 patient/caregiver health states in the model for DS. For health states for which no vignettes were developed (labeled A-C in **Table S8** and A-B in **Table S9**), utility values were estimated using the average values from 2 adjacent health states (1-2 for A; 3-4 for B; 5-6 for C).

Table S8. Vignettes and the LGS Economic Model Structure

No. of Drop Seizures	No. of Drop Seizure-Free Days		
	≤3	>3 to ≤15	>15
Seizure-free	N/A	N/A	Health state 1
≤45 seizures	N/A	Health state 2	State ‘A’ (no vignette)
>45 to ≤110 seizures	Health state 4	State ‘B’ (no vignette)	Health state 3
>110 seizures	Health state 6	State ‘C’ (no vignette)	Health state 5

Utility values from the vignette study were adjusted to match the health states used in the economic model for the Netherlands and were validated by Dutch clinical experts.

Table S9. Vignettes and the DS Economic Model Structure

No. of Convulsive Seizures	No. of Convulsive Seizure-Free Days		
	≤18	>18 to ≤24	>24
Seizure-free	N/A	N/A	Health state 1
≤8 seizures	N/A	Health state 2	State ‘A’ (no vignette)
>8 to ≤25 seizures	Health state 4	State ‘B’ (no vignette)	Health state 3
>25 seizures	Health state 6	Health state 5	N/A

Utility values from the vignette study were adjusted to match the health states used in the economic model for the Netherlands and were validated by Dutch clinical experts.

Caregiver vignettes were based on the corresponding patient vignettes. They included similar content describing the patient they cared for and an additional section describing the caregiver’s health and daily life. The patient vignettes were evaluated from the participants’ perspectives on having the condition. The caregiver vignettes described the perspective of someone who is 1 of 2 primary caregivers/parents of a 10-year-old child. Two caregivers were assumed because a DS caregiver survey indicated that most caregivers receive help with childcare from their partner and/or other family members.[2] The child was described as 10 years of age to avoid participants associating the caregiver burden with that of young children, while also selecting an age at which seizures are expected to be a significant contributing factor to the caregiver burden (based on clinician feedback on the natural history of the 2 conditions). The content of the final vignettes was informed by a limited, targeted review of quality-of-life publications in LGS and DS, interviews with DS caregivers, and interviews with LGS and DS clinical specialists.

Part 2: TTO Interviews

Members of the general public in the UK and Sweden were recruited using convenience sampling, including snowballing. Participants were eligible if they were adults (aged ≥18 years) and were not a caregiver/parent of someone with epilepsy. A total of 200 TTO interviews were conducted, which included 100 interviews with LGS patient and caregiver vignettes and another 100 interviews with DS patient and caregiver vignettes.

Participants were first asked to read the introductory text, which asked them to imagine they had the condition or that they were 1 of 2 primary caregivers/parents of a 10-year-old child with the condition. The conditions were not named and were referred to as ‘Epilepsy A’ (LGS) and ‘Epilepsy B’ (DS). The first exercise used a visual analog scale (VAS) ranging from 0 (worst possible state) to 100 (full health). Participants completed a TTO interview for the block of patient vignettes and another block of caregiver vignettes. Within each block, the vignettes were shuffled and the TTO was completed for each one in a quasi-random order. For each vignette, the interviewer recorded the utility value at the point of indifference. If participants rated any vignette as worse than dead, they were asked to confirm that they believed that this was the case. They then completed the lead-time TTO procedure for states worse than dead.

Analysis

The VAS ratings for each vignette were rescaled such that the value for the dead state was fixed at zero and all other values varied between 100 and the worse health state. The following formula was used to rescale the data:

$$V' = \left(\frac{\mathrm{V} - \mathrm{V}_{\mathrm{Dead}}}{100 - \mathrm{V}_{\mathrm{Dead}}}\right) * 100$$

where V′ is the rescaled VAS value, V is the original VAS value, and V_{Dead} is the value given to the dead state. After rescaling the VAS, data were summarized descriptively.

The TTO data were scored according to the point of indifference. The TTO data were summarized descriptively and presented as smoothed histogram distributions. For states in the economic models for which no vignettes had been evaluated, an average value was calculated based on the mean scores of 2 adjacent states.

REFERENCES

1. Kanters TA, Bouwmans CAM, van der Linden N, et al. Update of the Dutch manual for costing studies in health care. *PLoS One.* 2017;12:e0187477.
2. Lagae L, Irwin J, Gibson E, Battersby A. Caregiver impact and health service use in high and low severity Dravet syndrome: a multinational cohort study. *Seizure*. 2019;65:72-79.
